# The Crucial Role of Water Molecules in Stabilizing the Repressor/Operator Complex in the TrpR Repressor Family

**DOI:** 10.1063/4.0001116

**Published:** 2025-10-27

**Authors:** Andrzej Joachimiak, Youngchang Kim, Natalia Maltseva

**Affiliations:** 1 University of Chicago, Chicago, IL, USA; 2 Argonne National Laboratory, Lemont, IL, USA

## Abstract

Hydration plays critical role in protein structure, stability, dynamics and function. Recent advancements in high-resolution crystallography have provided a clearer understanding of the hydration patterns in proteins and their complexes with nucleic acids. The high level of detail allows to observe hydration patterns that were previously unresolved. Water-mediated specific protein interaction with DNA were initially discovered for *E. coli* the tryptophan repressor (TrpR) operator complex. Fixed water molecules are strategically positioned to mediate interactions between the repressor and the DNA bases. Here we report determination of high- resolution structures of TrpR and its complexes from *Francisella tularensis* including: aporepressor, complexes with L-Trp (activator), indole propionic acid (IPA) (inhibitor) and with specific *F. tularensis trp* operator. Structure analysis shows conservation of ligand binding and specific repressor-DNA interactions, including water-mediated and suggests that the majority of these interactions may be preserved across TrpR family. This research highlights the crucial role that water molecules play in mediating interactions between the repressor protein and the DNA bases that define the operator's identity. Bound water molecules are likely underrepresented in models of the interface of many specific biological complexes either because of spatial disorder in the crystal or inadequate resolution. These water molecules are not just passive participants; they are actively involved in the stereospecific recognition process, forming a dynamic and reversible network that extends the functional surfaces of the macromolecules involved. While this study focuses on the TrpR/operator system, the findings may have broader implications for understanding other protein-nucleic acid interactions and macromolecular complexes.

